# Tomato progeny inherit resistance to the nematode *Meloidogyne javanica* linked to plant growth induced by the biocontrol fungus *Trichoderma atroviride*

**DOI:** 10.1038/srep40216

**Published:** 2017-01-10

**Authors:** Hugo Agripino de Medeiros, Jerônimo Vieira de Araújo Filho, Leandro Grassi de Freitas, Pablo Castillo, María Belén Rubio, Rosa Hermosa, Enrique Monte

**Affiliations:** 1 Department of Microbiology and Genetics, Spanish-Portuguese Institute for Agricultural Research (CIALE), University of Salamanca, Salamanca, Spain; 2 Department of Phytopathology, Federal University of Viçosa, Viçosa Minas Gerais, Brazil; 3 Department of Crop Protection, Federal University of Pelotas, Pelotas, Rio Grande do Sul, Brazil; 4 Institute for Sustainable Agriculture, Spanish National Research Council (CSIC), Córdoba, Spain

## Abstract

Root-knot nematodes (RKN) are major crop pathogens worldwide. *Trichoderma* genus fungi are recognized biocontrol agents and a direct activity of *Trichoderma atroviride* (Ta) against the RKN *Meloidogyne javanica* (Mj), in terms of 42% reduction of number of galls (NG), 60% of number of egg masses and 90% of number of adult nematodes inside the roots, has been observed in tomato grown under greenhouse conditions. An *in vivo* split-root designed experiment served to demonstrate that Ta induces systemic resistance towards Mj, without the need for the organisms to be in direct contact, and significantly reduces NG (20%) and adult nematodes inside tomato roots (87%). The first generation (F1) of Ta-primed tomato plants inherited resistance to RKN; although, the induction of defenses occurred through different mechanisms, and in varying degrees, depending on the Ta-Mj interaction. Plant growth promotion induced by Ta was inherited without compromising the level of resistance to Mj, as the progeny of Ta-primed plants displayed increased size and resistance to Mj without fitness costs. Gene expression results from the defense inductions in the offspring of Ta-primed plants, suggested that an auxin-induced reactive oxygen species production promoted by Ta may act as a major defense strategy during plant growth.

The *Meloidogyne* species, commonly known as root-knot nematodes (RKN), are one of the most important groups of plant-parasitic nematodes worldwide^1^. This highly adapted obligate biotrophic polyphagous group of plant pathogens has a sedentary endoparasitic lifestyle with infective motile second-stage juveniles (J2) invading the plant near the tip of a root and migrating intercellularly through the tissue to the developing vascular cells. RKN establish an intimate relationship with their hosts, inducing the formation of the typical root gall, the primary visible symptom of RKN infection^2^. Some RKN pharyngeal gland secretions act as effectors, which serve to subvert the defense system while modifying the host’s cell functions^3^.

RKN migrate in a stealthy way between the root cells, avoiding plant defense responses along their migration path and manipulating defense pathways in the root galls to promote infection of the tissue^2^,^4^. Temporary local defense suppression, with early strong attenuation of the salicylic acid (SA)- and ethylene (ET)-dependent defense pathways, has been observed in the feeding sites of RKN-infected plants^2^,^5^,^6^,^7^. Such suppression is accompanied by reduced expression of SA-biosynthesis genes^8^ and SA-responsive pathogenesis-related (PR) protein genes^6^,^9^ in aboveground tissues. The JA pathway has been proposed as the key defense against RKN, being efficiently activated and modulated by ET and brassinosteroids^10^. Although it could be thought that SA is only moderately involved, slight increases in SA levels could be sufficient enough to heighten plant defenses against RKN in tomato^11^, since the repression of host defense SA signaling has been associated with the successful development of RKN^12^.

Nematode management has often been based on the application of nematicide molecules. In spite of its reasonable efficacy, the use of nematicides has been a growing concern because of various environmental aspects, risks to human health and high cost for growers^13^. Studies exploring the use of antagonistic and competitive microorganisms against RKN have gained great attention^14^, mainly because of their ability to induce local and systemic defenses in different plants^15^,^16^,^17^. Among them, fungal and oomycete biocontrol agents from the genus *Trichoderma* and their hydrolytic enzymes^18^ have demonstrated biocontrol potential against RKN, including egg laying and hatching^19^,^20^,^21^,^22^. In addition to the direct action against their prey, some *Trichoderma* rhizosphere-competent strains have been shown to have direct effects on plants, increasing their growth and nutrient uptake, percentage and rate of seed germination, and stimulation of plant defenses against biotic and abiotic damage^23^,^24^,^25^. It has been described that *T. harzianum* increases enzymatic activities in tomato leading to a systemic resistance against the RKN *Meloidogyne javanica* (Mj)^22^,^26^. It has also been reported that RKN attack involves a consistent downregulation of the SA-dependent systemic defense *PR1* gene in tomato roots^27^, but such downregulation seems to decrease over time in *T. harzianum* pre-treated plants. In contrast, RKN attack involves the upregulation of the JA/ET response factor *JERF3* that was markedly stronger in roots pre-treated with *T. harzianum*. A recent study has shown that *T. harzianum*-induced systemic resistance against *Meloidogyne incognita* in tomato adapts the priming of SA- and JA-related defense responses according to the stage of the dynamic RKN infection cycle^28^.

Our study aimed to evaluate the significance of the plant-mediated signals of *T. atroviride* (Ta)-colonized roots that prime defense responses against Mj attack. We have applied an experimental design to obtain information about the systemic defense responses unequivocally triggered by *Trichoderma* in tomato using a split-root system^16^,^28^,^29^ in which both organisms, Ta and Mj, can be separately co-inoculated. In addition, the heritability of traits related to defense systems, induced by Ta against Mj in tomato, was explored in F1 progeny.

## Results

### Biocontrol of Ta against Mj in tomato

**Figure 1:**
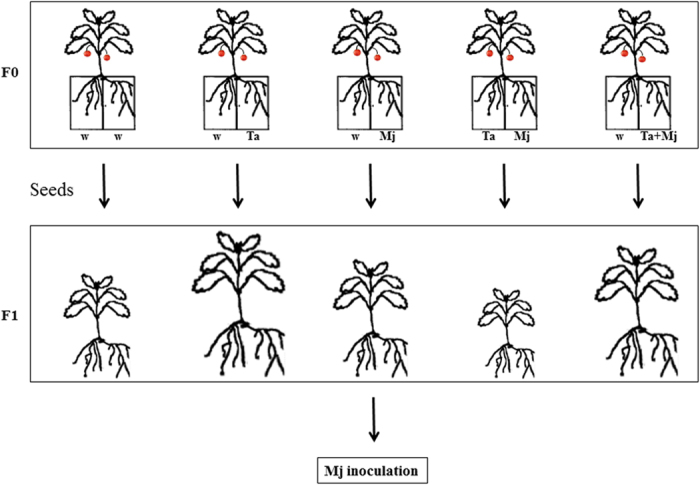
Experimental procedure used for *in vivo* analysis of systemic defense in tomato plants. Five-week-old tomato plants (F0) were transplanted following a split-root system designed experiment and treated with *Trichoderma atroviride* T11 (Ta) and/or *Meloidogyne javanica* (Mj) in the following way: i) (w/w) the two root system halves treated with sterile water (control); ii) (w/Ta) one root system half treated with Ta; iii) (w/Mj) one root system half treated with Mj; iv) (Ta/Mj) one root system half treated with Ta and the other half with Mj; and v) (w/Ta + Mj) one root system half with treated with Ta plus Mj. F1 plants, derived from the corresponding F0 treatment, were inoculated with Mj when they were 21-days old. See Methods for more details.

**Figure 2:**
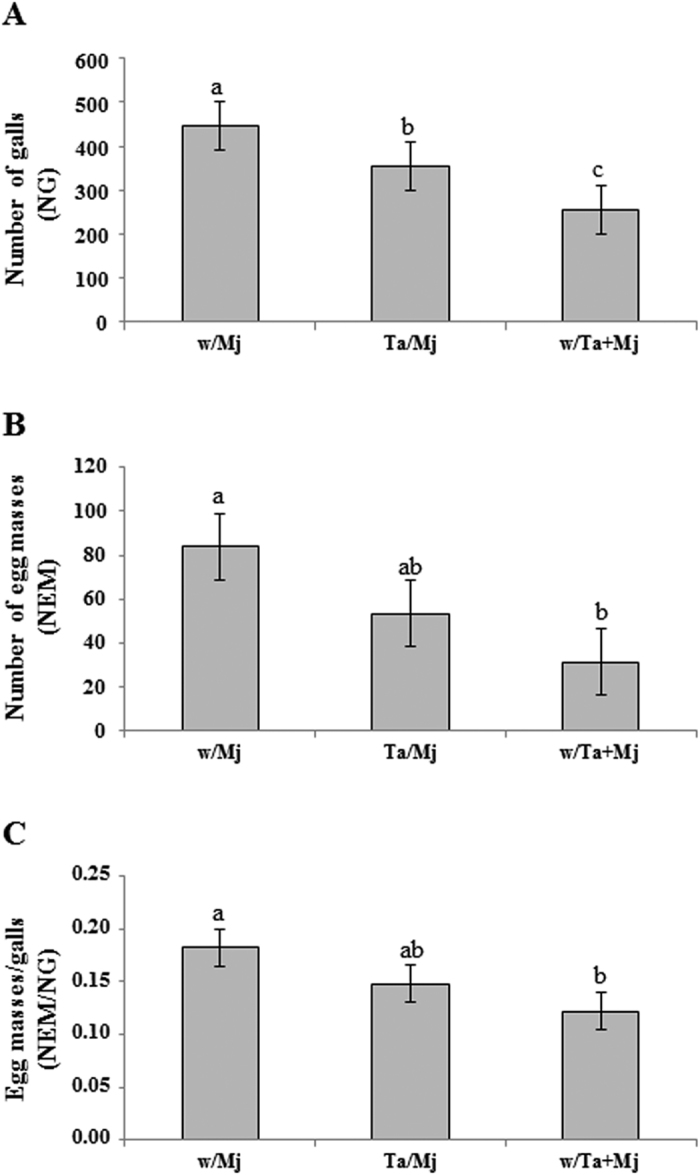
*In vivo* biocontrol ability of *Trichoderma atroviride* T11 (Ta) against *Meloidogyne javanica* (Mj). (**A**) Number of galls (NG), (**B**) Number of egg masses (NEM), and (**C**) NEM/NG ratio calculated at 30 days after infection with Mj when tomato plants were six-weeks old. The split-root treatments were as indicated in the legend of Fig. 1. Data of three plants per treatment from two independent experiments (n = 6) were submitted to *ANOVA* and the comparison of means by Tukey test (*P* < *0.05*) carried out. Bars with different letters indicate statistically significant differences between the different treatments.

Using a tomato split-root experimental design, the biocontrol potential of the Ta strain T11 was evaluated (Fig. 1) systemically, two root halves separately inoculated with Ta and Mj (Ta/Mj treatment), and in direct confrontation (w/Ta + Mj) against the RKN Mj. Parameters related to Mj attack, such as the number of galls (NG), the number of egg masses (NEM) and the NEM/NG ratio are shown in Fig. 2, where differences within these variables were observed (*P* < *0.05*) between the infection control w/Mj and the w/Ta + Mj treatment plants. Plants in which Ta and Mj were directly confronted showed reductions of 42% NG, 60% NEM and 35% NTNR. In addition, the NG value was significantly lower (20%) with the Ta/Mj treatment compared to that of w/Mj, showing that Ta induces systemic resistance to Mj, resulting in the development of fewer feeding sites. Nevertheless, the induction of defenses did not seem to affect Mj reproduction since NEM and NEM/NG values in Ta/Mj were not significantly different to those in w/Mj.

**Figure 3:**
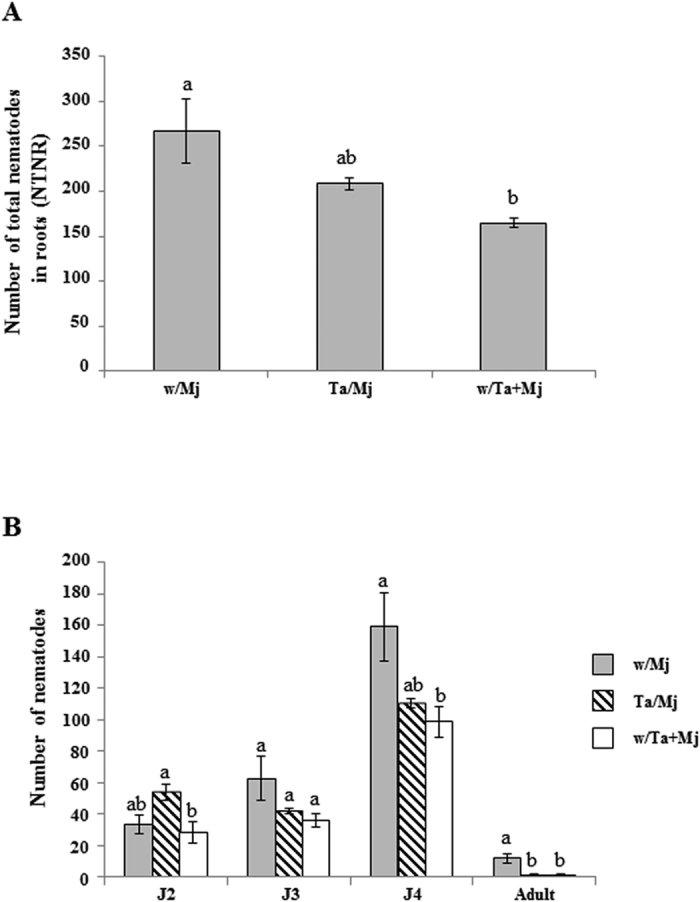
Resistance induced by *Trichoderma atroviride* T11 (Ta) against *Meloidogyne javanica* (Mj) in tomato plants. (**A**) Number of total nematodes in roots (NTNR) and (**B**) Number of nematodes corresponding to different developmental stages (J2, second stage juvenile; J3, third stage juvenile; J4, fourth stage juvenile; and adult). NTNR and number of nematodes calculated at 15 days after inoculation with Mj when tomato plants were six-week old. The split-root treatments were as indicated in the legend of Fig. 1. Data of four plants per treatment from two independent experiments (n = 8) were submitted to *ANOVA* and the comparison of means by Tukey test (*P* < *0.05*) was carried out. Bars with different letters indicate statistically significant difference between the different treatments.

To explore the biocontrol effect of Ta against Mj we determined the number of total nematodes inside the roots (NTNR) and the number of juveniles J2, J3 and J4, and adults in tomato plants 15 days after inoculation (dai) with 300 J2 units (Fig. 3). Lower NTNR numbers were registered for w/Ta + Mj treatment (Fig. 3A) and a progressive increase of juvenile developmental stages was observed in the w/Mj treatment (Fig. 3B). Moreover, Ta reduced the number of J4 and adults (*P* < *0.05*) in w/Ta + Mj plants, and the number of adult nematodes was also significantly reduced (87%) in the Ta/Mj treatment. Gall and adult numbers support that Ta triggers a systemic defense response against Mj in tomato even without establishing contact with the nematode.

### *Trichoderma* induces systemic defenses to RKN in tomato

**Figure 4:**
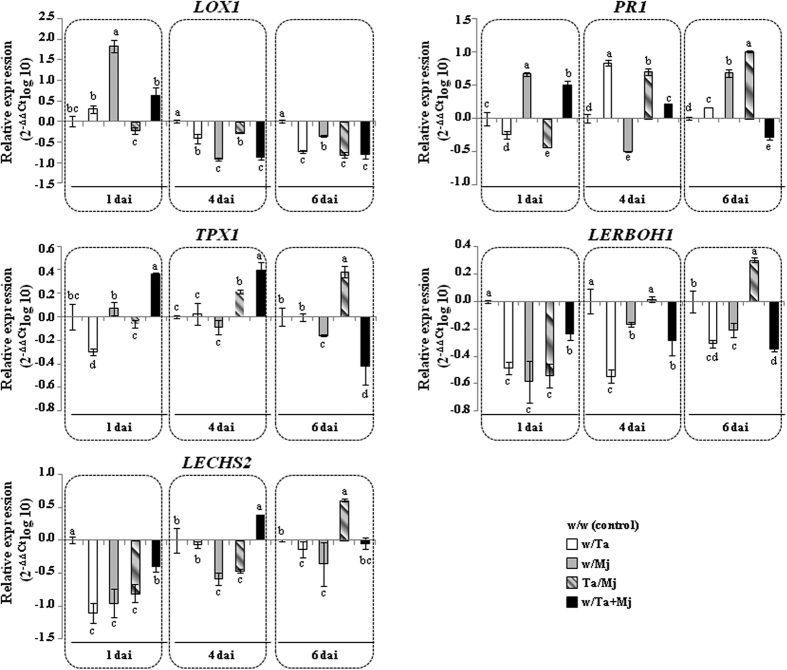
Defense marker gene expression patterns in tomato roots in response to *Trichoderma atroviride* T11 (Ta) and/or *Meloidogyne javanica* (Mj). Total root cDNA was subjected to qPCR to quantify *LOX1, PR1, TPX1, LERBOH1* and *LECHS2* gene expression related to different plant defense responses at 1, 4 and 6 days after inoculation with Mj. The actin gene was used as a control. Values correspond to relative measurements against the respective control conditions (w/w) (2^−ΔΔ*CT*^ = 1) for each time point, and are expressed as log_10_. Error bars represent standard deviations of the mean values for three technical replicates of cDNA from two independent experiments. Each cDNA was obtained from a root-pool of three different F0 plants for each treatment and time point. The levels of expression were tested using one-way analysis of variance (ANOVA) followed by Tukey’s test. For each gene and time, different letters represent significative differences (*P* < 0.05).

The roots of the tomato plants from the split-root experiment were collected for carrying out a quantitative Real-time PCR (qPCR) expression study of defense-related genes. To analyze how and to what extent Ta induced systemic defense against Mj in tomato, as observed in the significant reduction of NG and adult nematodes in roots from Ta/Mj treatment (Figs 2 and 3), the expression of genes encoding lipoxygenase 1 (LOX1), pathogenesis-related protein 1 (PR1), a cell wall peroxidase (TPX1), a NADPH oxidase (LERBOH1) and chalcone synthase 2 (LECHS2) was monitored at three sampling times. Ta and Mj were applied to the plants at different time points, in which Ta inoculation was performed 5 days previous to that of Mj, except in the w/Ta + Mj treatment. All of the data from all treatments corresponded to 1, 4 and 6 dai with Mj as shown in Fig. 4.

Compared to the control (w/w), only tomato roots from the w/Mj treatment displayed an increased *LOX1* expression level at 1 dai, this gene being downregulated in roots of all treatments at 4 and 6 days. Conversely, *PR1* showed an opposite expression profile to that of *LOX1* in w/Ta roots at the three different time points considered. However, antagonism between JA- and SA-defense pathways was not observed in w/Mj roots at 1 and 4 dai, since *PR1* was also upregulated at 1 dai and transiently downregulated at 4 dai. In general, the expression profiles of *LOX1* and *PR1* detected in Ta/Mj roots were similar to those from the w/Ta treatment except for *LOX1* at 1 dai and *PR1* at 6 dai. The *LOX1* expression profile detected in w/Ta + Mj roots was similar to that in w/Mj roots, nevertheless the concerted regulation of *PR1* was only detected in both treatments at 1 dai. A decrease of *PR1* expression was observed in w/Ta + Mj roots over time; the gene was only temporarily downregulated in w/Mj and appeared upregulated at 6 dai.

In general, the *TPX1* expression profile was similar to that of *PR1* in w/Ta at 1 dai, in Ta/Mj at 4 and 6 dai, and in w/Ta + Mj roots at the three time points. A downregulation of *LERBOH1* and *LECHS2* was observed in w/Ta and w/Mj treatments at the three time points, and also in Ta/Mj roots at 1 dai, while these two genes were upregulated upon Ta/Mj treatment at 6 dai.

### Heritability of the induced defense by Ta against Mj in tomato

**Figure 5:**
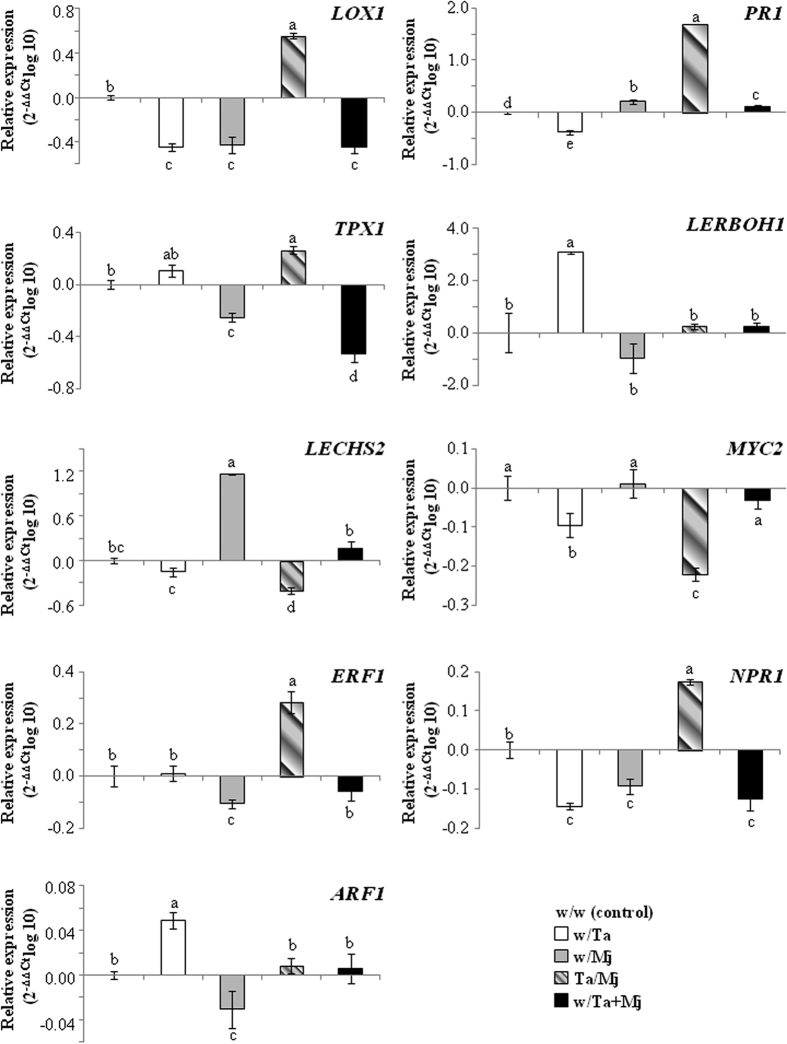
Defense gene expression in *Trichoderma atroviride*-primed tomato plant progeny. Total RNA extracted from two-week-old F1 plants, derived from split-root tomato plants subjected to the five treatments indicated in Fig. 1. cDNA was subjected to qPCR to quantify *LOX1, PR1, TPX1, LERBOH1, LECHS2, MYC2, NPR1, ERF1* and *ARF1* gene expression. The actin gene was used as a control. Values correspond to relative measurements against the respective control condition (w/w) (2^−ΔΔ*CT*^ = 1) and are expressed as log_10_. Error bars represent standard deviations of the mean values for three technical replicates of cDNA from two independent experiments. Each cDNA was obtained from ten different F1 plants for each treatment. The levels of expression were tested using one-way analysis of variance (ANOVA) followed by Tukey’s test. For each gene different letters represent significative differences (*P* < 0.05).

The expression levels of seven marker genes were analyzed in 15-day-old F1 plants coming from seeds collected from the five split-root tests (Fig. 5). A downregulation of *LOX1* was detected in F1 plants from all treatments except for plants coming from Ta/Mj seeds. *PR1* was only downregulated in w/Ta F1 plants and those from the Ta/Mj treatment showed the highest *PR1* expression levels. The *PR1* upregulation detected in these plants was accompanied by increased expression of *LOX1* and *TPX1*. This result would be indicating that these F1 plants, derived from already primed plants in a SA-dependent manner, were capable of initiating JA- and SA-dependent defense priming. Regarding *LERBOH1*, only increased expression was detected in w/Ta F1 plants. In general, the results for *LECHS2* and *TPX1* in F1 plants showed an opposite gene expression profile. Phytohormone crosstalk regulation in F1 plants was explored by gene expression analysis of the TFs MYC2 and ERF1 that lead to the transcription of JA responsive genes in an antagonistic manner, NPR1 that controls the onset of SA-dependent systemic acquired resistance, and ARF1 that regulates auxin responses (Fig. 5). *MYC2* and *ERF1* genes were respectively downregulated in w/Ta and w/Mj F1 plants. The lowest *MYC2* expression levels were detected in Ta/Mj F1 plants, while they showed the highest levels of expression for *ERF1*; thus, indicating a prevalence of Ta over Mj in the plant transcriptional signaling. As expected for a SA-responsive gene activator, the upregulation of *NPR1* was accompanied by an expression increase of *PR1* in Ta/Mj F1 plants. The similar *ARF1* and *LERBOH1* expression patterns, detected in all F1 plants, imply that both genes can be co-regulated, suggesting an auxin-induced reactive oxygen species (ROS) production. In parallel, opposite *ARF1* and *MYC2* expression patterns were detected. *ARF1* upregulation in w/Ta F1 plants was consistent with the detected *LOX1* and *MYC2* downregulation, components of a regulatory pathway at the crosstalk of auxin and JA.

**Figure 6:**
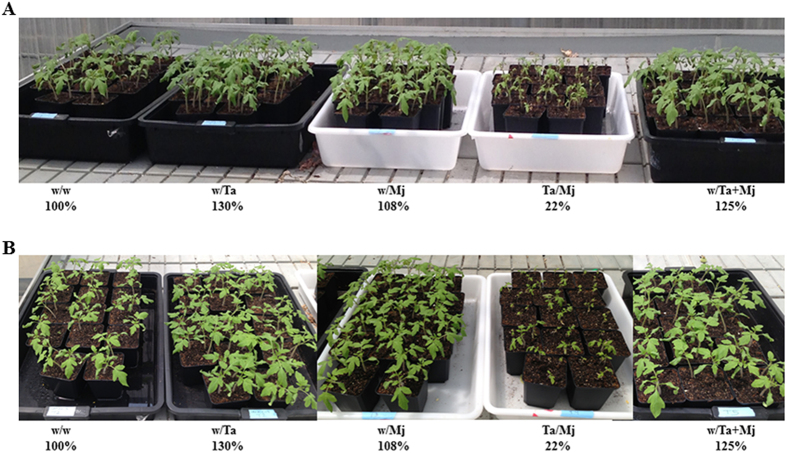
Phenotypical differences among *Trichoderma atroviride*-primed tomato plant progeny. (**A**) Frontal view of two-week-old F1 plants, derived from seeds collected from split-root tomato plants subjected to the five treatments indicated in the legend of Fig. 1. (**B**) The same plants viewed from above. Green mass percentages are referred to that of the control plants (w/w = 100%) and obtained from five plants per treatment of two independent experiments.

**Figure 7:**
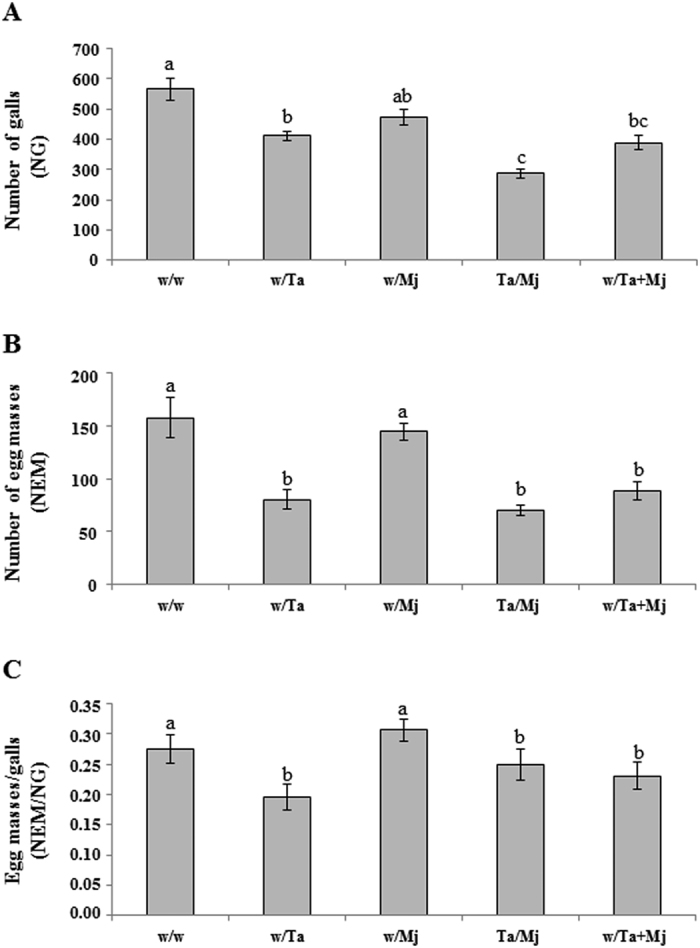
Transgenerational biocontrol against *Meloidogyne javanica* (Mj) in *Trichoderma atroviride*-primed tomato plant progeny. (**A**) Number of galls (NG), (**B**) Number of egg masses (NEM), and (**C**) NEM/NG ratio calculated in 51-day-old F1 plants at 30 days after infection with Mj, when plants were 21-days old. These plants were derived from seeds collected from split-root tomato plants subjected to the five treatments indicated in the legend of Fig. 1. Data from five plants per treatment of two independent experiments (n = 10) were submitted to *ANOVA*, and the comparison of means by Tukey test (*P* < *0.05*) was carried out. Bars with different letters indicate statistically significant differences among treatments.

F1 tomato plants from the five split-root tests showed phenotypic differences among them when they were grown under greenhouse conditions (Fig. 6). The green mass of 2-week-old F1 plants was calculated, and the value of 100% was assigned to the green mass coming from the w/w F1 plants (control). F1 plants derived from w/Ta and w/Ta + Mj treatments showed the highest green mass values of 130 and 125%, respectively; the green mass value for w/Mj reached 108%. However, F1 plants from the Ta/Mj treatment exhibited a dramatic reduction in size with a green mass of only 22%. Differences in canopy and levels of development were also observed among the F1 plants derived from the five split-root treatments at 30 dai with Mj (Fig. S1). Compared to the w/w control condition, the Mj-infected F1 plants from w/Ta treatment increased their aboveground size by 15%, and all of the descendants from Mj-challenged plants showed a reduced size. At 30 dai with Mj, a reduction of 20, 40 and 10% in green mass value was detected for w/Mj, Ta/Mj and w/Ta + Mj F1 plants, respectively. Furthermore, w/w and w/Mj F1 plants showed the highest NG, NEM and NEM/NG average values, while those plants coming from the seeds of the three Ta treatments gave significantly lower numbers (Fig. 7). In addition, no significant differences for the NEM value and NEM/NG ratio were observed among w/Ta, Ta/Mj and w/Ta + Mj F1 plants (Fig. 7B and C).

## Discussion

As expected, in the present study the simultaneous root application of Ta and Mj significantly reduced NG and NEM values and the NEM/NG ratio, indicative of a direct biocontrol activity against Mj as previously reported in *T. harzianum*^20^,^21^. When *Trichoderma* and RKN were separately co-inoculated using a split-root system (Ta/Mj treatment), these plants also presented a significantly reduced NG and number of nematodes in their adult stage; therefore demonstrating that Ta may induce systemic resistance towards Mj, as it has been recently reported for *T. harzianum* against *M. incognita* in tomato^28^. This result may be attributed to the interference of pathogen migration into the roots, as well as to the difficulty in establishing feeding sites, once the nematode stages of development and the NG values indicate a delay in the nematode life cycle and a reduction of gall formation, respectively. However, the systemic resistance induced by Ta did not interfere with the nematode penetration or nematode reproduction, as was indicated by the NTNR values and NEM/NG ratios, respectively. In this experiment Mj was added to the substrate 5 days after in Inoculation with Ta but it would be expected that a longer time frame between Ta and Mj inoculation could lead to a significant control of nematode penetration and reproduction, as observed in tomato roots inoculated with *M. incognita* eggs three weeks after the application of *T. harzianum*^28^. The resistance response of tomato roots, expressed by a lower NG and NEM values and the corresponding NEM/NG ratio of Mj (Fig. 2), in the treatment w/Ta + Mj could be related to the nematicide activity of Ta and the direct competition between Mj (for the feeding site) and Ta (for an endophytic growth in the apoplast) in the same half of the split root, since Ta and Mj were simultaneously inoculated. The contrasting results observed for NTNR and infective J2 accumulation between the treatments w/Mj and Ta/Mj (Fig. 3) can be due to the systemic effect of Ta that would be interfering the development of the nematode, since higher J3, J4 and adult nematode numbers were recorded for the w/Mj treatment.

The expression profiles detected for *LOX1* and *PR1* in the w/Ta treatment are in agreement with the mutual antagonism between the JA and SA, previously observed in *Trichoderma*-tomato interactions^30^. The induced resistance triggered by Ta indicates that the JA and SA phases act concertedly and the transient JA signaling peak, observed in the first 24 h, can represent residual levels of the early initiation phase of resistance^31^, as expected in plants analyzed at 6 days after Ta application. When the JA signaling is low enough, SA contributes to subsequent events in establishing systemic immunity. The SA undulated defense response can be explained by the temporal phytohormone transcription cascades that are not necessarily active simultaneously^32^.

The transient downregulation of *LOX1* and *PR1* detected in w/Mj plants at 4 dai is in agreement with previous observations in which RKN seemed to initially induce the JA- and SA-dependent pathways; although, parts of these pathways were very quickly repressed again^8^. A temporary attenuation of SA-responsive defense has also been observed in the feeding site initiation of RKN-infected plants^2^,^5^,^6^,^7^, later followed by activation in the gall tissue^2^, and systemic transmission of *PR1* undulated expression^7^,^9^. Since the JA pathway is the major defense strategy against RKN^4^, its attenuation at 4–6 dai in w/Mj treatment could explain the high values related to the level of infection registered in these plants (Figs 2 and 4).

The significant reduction of NG and the number of adult stage nematodes detected in the Ta/Mj treatment would not only be supported by the upregulation of *PR1* at 4 and 6 dai with respect to w/Mj but by the upregulation of *TPX1* at these two time points (Figs 3 and 4). This last gene is involved in the formation and deposition of lignin in the tomato cell wall^33^, and its repression has been described in tomato-RKN compatible interactions^7^. The *PR1* and *TPX1* downregulation observed at 24 h in w/Ta and Ta/Mj tomato roots agrees with the transient repression in *Arabidopsis* of SA-dependent immune responses to allow *Trichoderma* early root colonization^34^, although a subsequent SA-mediated reactivation of defenses is necessary to avoid root invasion by *Trichoderma*^35^. Interestingly, the similar *PR1* time course expression profile observed in w/Ta and Ta/Mj treatments (Fig. 4) could indicate that Ta-induced defenses might take predominance over the response activated by the pathogen. It has been proposed that *T. harzianum* primes tomato root tissues for faster SA-regulated defense responses to protect roots against RKN invasion^28^.

The *PR1* and *LOX1* expression profiles detected in roots from w/Ta + Mj treatment over time show that marker genes of the JA and SA pathways overlap when the biocontrol agent was in direct contact with the nematode. This observation is in agreement with the previously reported *PR1* downregulation induced by *T. harzianum* pre-treatment in tomato roots infected with Mj in the same pot^27^; although, we could not detect the increase of the JA/ET-mediated defense in w/Ta + Mj roots as observed in that study. The attenuated *PR1* and *LOX1* expression levels detected in w/Ta + Mj plants at 1 dai compared to those of the treatment w/Mj, could be due to the direct nematicide activity of the Ta strain used in our study, resulting in lower levels of Mj. The transient expression of *PR1* in w/Ta + Mj plants from 1 to 6 days would be indicating an undulated defense response. However, it is difficult to propose a model of hormonal crosstalk in plants occurring under multi-attacker interactions^36^.

Nematodes utilize the ROS depletion caused by the loss of NADPH oxidase activity, and the local suppression of flavonoid biosynthesis, in their own benefit to migrate and initiate the establishment of the feeding sites^37^,^38^. Furthermore, the chalcone synthase *LECHS2* gene, involved in accumulation of flavonoid and isoflavonoid phytoalexins, components of the SA defense pathway, was suppressed at 24 h in aboveground parts of tomato plants root-colonized by *T. harzianum*^34^. Thus, the downregulation of *LERBOH1* and *LECHS2* detected in w/Ta and w/Mj plants could facilitate root colonization by both organisms (Fig. 4). As observed in the *PR1* and *TPX1* expression profiles from Ta/Mj plants, *LERBOH1* and *LECHS2* showed upregulation in this treatment over time, providing evidence that the systemic defense induced by Ta leads to the observed significant reduction of NG and adult nematodes (Figs 2A and 3B). Interestingly, *LERBOH1* and *LECHS2* marked a hypersensitive response when they were upregulated upon Mj formation of gall tissue in the Ta/Mj treatment at 6 dai, a result that was also in agreement with the SA-dependent priming detected in Ta/Mj plants.

Independent of the application method, all descendants of Ta-treated plants retrieved at 30 dai with Mj showed control against the nematode (Fig. 7), with significant lower NG and NEM values and NEM/NG ratio with regard to the control w/w F1 plants. In addition, the best protection corresponded to the Ta/Mj treatment F1 plants, specifically those with the smallest size before and after infection with Mj (Figs 6 and S1). These results indicate that the first generation from Ta-primed tomato plants does not lose its protected state, and exhibits enhanced resistance to RKN, as previously reported in bacteria-*Arabidopsis* biotrophic interactions^39^,^40^. Moreover, plant growth promotion induced by *Trichoderma* was inherited; this beneficial effect was exhibited at varying degrees depending on which treatment the plant was derived. Thus, the *Trichoderma* switching signatures involved in balancing the plant’s defense and growth^41^ seem to be inherited in a Ta treatment-dependent manner. In agreement with the fitness costs associated with induction of defenses, our study confirms that the Ta-induced plant growth promotion inherited by w/Ta F1 plants was accompanied by significant reduction of defenses dependent on the SA and JA pathways (Fig. 5). The *MYC2* downregulation, without activation of the *ERF1*-branch of the JA response pathway, observed in w/Ta F1 plants is in agreement with that of *LOX1* and the increased green mass observed in these plants (Figs 5 and 6). Since *MYC2* negatively regulates auxin biosynthesis and lateral root growth^42^, its downregulation would foster auxin production and the increased size observed in w/Ta F1 plants. Similarly the downregulation of *NPR1* and *PR1* is an indicator that SA is not accumulated in w/Ta offspring, at least at a concentration capable of inducing *NPR1-*dependent *PR1* gene expression^43^. A reduction of JA- and SA-dependent defenses would irretrievably expose the plants to any pathogen attack. However, w/Ta F1 plants also showed that *LERBOH1* and *TPX1* were concertedly upregulated with the auxin response factor ARF1, and increased resistance against Mj. Continuous defense signal amplification would waste energy and the *NPR1* and *PR1* downregulation associated to auxin-induced ROS production observed in w/Ta F1 plants is in agreement with the proposed antagonistic relationship between SA and apoplastic ROS signaling that regulates defense gene expression in plants^44^. Our results agree to the proposed model in which accumulation of auxin in cells is not only linked to plant growth control but to altered cellular redox status, involving the production of an oxidative burst through the activation of NADPH oxidases and cell wall peroxidases, to provide an appropriate defense response^45^. Oxidative burst imbalance can be regulated by the activation of the antioxidant machinery that appears to be a general mechanism triggered by *Trichoderma* in different plant species (*Arabidopsis*, cucumber and tomato) to enhance tolerance to a range of abiotic stresses^23^. Although a better understanding of ROS formation and the oxidative burst function as a consequence of plant-*Trichoderma* interaction is needed, our results suggest that Ta-primed plant offspring displays increased resistance to Mj without fitness costs, and the auxin-induced ROS production promoted by Ta could be directly involved in cell wall loosening and cell elongation^46^, acting in turn as a major defense strategy when the plant balance is tipped towards growth. The low Mj resistance detected in w/Mj F1 plants indicates that the descendants from plants challenged with Mj were not primed for defenses. The upregulation of *PR1* and *LECHS2* would indicate a SA-mediated induction of phytoalexin production in a NPR1-independent manner. Although, it has been reported that an accumulation of flavonoids caused by chalcone synthase activity may block auxin transport^47^, the up- and downregulation of *LECHS2* and *ARF1,* respectively, detected in 2-week-old-F1 plants derived from w/Mj treatment was not accompanied by a reduction of their green mass. However, these w/Mj F1 plants presented lower green mass values than the control at 30 dai with Mj although no differences in NG, NEM and NEM/NG parameters were detected (Figs 7 and S1).

Since Ta positive growth effect was not reflected in the increased size of F1 plants coming from Ta/Mj treatment, defense responses would prevail over growth promotion as the inherited trait in plants challenged with Ta and Mj. Special attention must be paid to the simultaneous upregulation of *PR1, LOX1* and *TPX1* in F1 plants coming from Ta/Mj seeds. The marked upregulation of *PR1* could indicate resistance to RKN considering that *PR1* was highly induced in shoot upon RKN attack in resistant tomato plants^12^. The increased JA- and SA-dependent defenses detected in these plants agree with the upregulation of *ERF1* and *NPR1* and also with the enhanced resistance to Mj attack. Cooperation and sequential positive interactions have also been reported between SA and JA/ET pathways^48^. Moreover, the transgenerational Ta priming showed a similar activation of the SA-, JA-, and ET-regulated defense pathways as described in incompatible RKN-tomato interactions^11^. Interestingly, an approximately 5-fold reduction of green mass was detected in 2-week-old Ta/Mj F1 plants compared to that of the control F1 plants (Fig. 6); although, only a 1.6-fold reduction was detected in these plants at 30 dai with Mj (Fig. S1). These data indicate that the growth of Ta/Mj F1 plants was not negatively affected by Mj infection or, at least, the observed deleterious growth was less evident than that caused by the pathogen in the rest of F1 plants. It is not easy to draw conclusions on a system with two simultaneous plant invaders, especially if one of them becomes a prey for the other. In w/Ta + Mj F1 plants, the responses to Ta and Mj were assessed, and also the reaction to the potential damage associated with the molecular patterns released as a result of the hydrolytic activity of Ta on Mj. In the case of 2-week-old w/Ta + Mj F1 plants in absence of the pathogen, the expression profiles of defense marker genes and the growth degree were similar to those obtained for w/Mj F1 plants. However, the w/Ta + Mj F1 plants challenged with Mj showed increased resistance at the expense of their fitness. Results shown in Fig. 7 indicate that F1 plants from the three Ta treatments inherited resistance against Mj, but the gene expression profiles observed in these F1 plants indicate that the defense induction occurred through different mechanisms in varying degrees depending on the Ta-Mj interaction.

Ta symbiosis and Mj infection are expected to induce epigenetic changes by guiding DNA methylation and chromatin modifications in the plant. In the case of RKN, genes involved in these activities and small RNA formation were induced inside the giant cells^2^,^7^. However, the underlying mechanisms that support the beneficial activity of *Trichoderma* to plants, in terms of heritable growth and defense, have yet to be deciphered. Nevertheless, the heritable key signaling nodes that emerged from favorable plant-*Trichoderma* interactions are targets that should be taken into account in further studies to understand why the *Trichoderma*-regulated fitness costs associated with induction of defenses depend not only on the Ta application itself but in the way that Ta and the pathogen are or are not confronted.

In addition to a direct biocontrol activity against Mj, we report on the capacity of Ta inducing systemic resistance to RKN without establishing any contact with the pathogen, as well as on the heritability of this priming for enhanced resistance and growth promotion without compromising the level of resistance of the plant’s offspring to the nematode.

## Methods

### Organisms used

The RKN *Meloidogyne javanica*, referred to as Mj, was used throughout the present work. Mj was collected from infected tomato roots in Córdoba (Spain), and cultured from a single egg mass. The species identification was carried out integrating morphological approaches based on females, males and J2s^49^, α-esterase isoenzyme phenotypes^50^ and molecular analysis of cytochrome oxidase II^51^. For Mj sterilization, nematode eggs were placed on a sterile Whatman filter holder (GE Healthcare Europe GmbH, Barcelona, Spain) containing a cellulose acetate filter membrane with a 5 μm pore size (GE Healthcare Europe GmbH). Then, the eggs on the filter were exposed to 0.01% (w/v) mercuric chloride (Hg_2_Cl_2_) for 10 min (Sigma-Aldrich, Madrid, Spain), followed by 0.7% streptomycin solution (Sigma-Aldrich) and three washing steps with 50 mL of sterile distilled water^52^. Sterile eggs were then collected from the membrane and placed in sieves with a 25-μm pore size in 0.01 M 2-(N-morpholino) ethanesulphonic acid (MES) buffer (Sigma-Aldrich) under aseptic dark conditions for 3 days, J2s were collected after hatching.

*Trichoderma atroviride* (Ta) IMI 352941 (International Mycological Institute, CABI Bioscience, Egham, UK), also referred to as strain T11, was the fungus used in the greenhouse assays. This strain was grown on potato dextrose agar medium (PDA, Difco Laboratories, Detroit, USA) and the spores were stored in 30% glycerol at −80 °C. Spores from 7-day-old PDA plates were harvested by adding 5 mL of sterile water to the plates and by scraping the culture with a rubber spatula. These suspensions were filtered through a double layer of cheesecloth to separate large mycelial fragments from conidia. Spore concentrations were calculated using a counting chamber and the suspension indicated below was applied to tomato seeds.

Tomato seeds (*Solanum lycopersicum* “Marmande”) were sterilized in 70% ethanol for 10 min and in sodium hypochlorite for 10 min. Later, seeds were washed thoroughly in sterile distilled water before being used.

### Biocontrol and induction of tomato defense assays

Sterilized tomato seeds were sown in 3 L pots containing commercial organic substrate Tref (Jiffy, Castillo Arnedo, Navarrete, Spain), previously autoclaved at 121 °C for 1 h on two successive days. The pots were cared for in a greenhouse at 22 ± 4 °C, and watered appropriately. After five weeks tomato plant roots were split into two halves and each root portion was planted into a pot containing a mixture of Tref and vermiculite, previously autoclaved as described above, in a proportion of 3:1. The split-root experimental design consisted of five treatments: i) the two root system halves treated with sterile water (w/w) was the control condition; ii) one half of the root system inoculated with Ta (w/Ta); iii) one half of the root system inoculated with Mj (w/Mj); iv) one half of the root system inoculated with Ta and the other half inoculated with Mj (Ta/Mj); and v) one half of the root system inoculated with Ta plus Mj (w/Ta + Mj) (Fig. 1). One week after transplanting, a root portion of each tomato plant was inoculated with 1 mL of T11 conidial suspension (1 × 10^7 ^spores/mL) in w/Ta and Ta/Mj treatments or 1 mL of T11 conidial suspension (1 × 10^7 ^spores/mL) plus 500 units of J2 in w/Ta + Mj treatment. Mj infection was performed with 500 J2s in treatments w/Mj and Ta/Mj 12 days after transplanting. Greenhouse assays were repeated twice in a completely randomized design, and 15 replica plants were included for each treatment. Three root samples from three different replica plants per treatment were collected at 1, 4 and 6 dai with Mj for gene expression analysis by qPCR. Roots were washed, immediately frozen in liquid nitrogen and stored at −20 °C until used. At 30 dai with Mj, three root samples from three other different replica plants of w/Mj, Ta/Mj and w/Ta + Mj treatments were collected to estimate the NG and the NEM as previously described^53^. These data were used to calculate the NEM/NG ratio.

### Nematode penetration and developmental stage assay

This assay was set up with the split-root experimental design described above, although minor changes were made. Plant substrate was inoculated with 300 units of J2, and penetration rates and life developmental stages were determined at 15 dai with Mj. Four root samples from four different plants of the treatments w/Mj, Ta/Mj and w/Ta + Mj were collected and stained using the fuchsin acid method^54^. The total number of nematodes in root (NTNR) and developmental stages J2, J3, J4 and adult were estimated in two independent biological experiments, as previously described^55^.

### Heritability of induced defense responses

Three plants (F0) from every treatment were maintained in the greenhouse until fruit production. Two fruits per plant from independent greenhouse experiments were gathered to generate five different seed pools (Fig. 1). Seeds were washed, sterilized as described above, and sown into 500 mL pots containing the commercial substrate Tref, autoclaved as already described. The pooled seeds were used in two independent experiments. In every experiment, three seeds were sown per pot and 14 pots, with a total of 42 F1 plants, were prepared per treatment. At 14 days, five F1 plants per treatment were used to calculate the geometric parameters of height and width of the green mass using the following methodology. The height of the canopy was measured perpendicular to the soil, and because the width of the canopy varied with plant height, three 1/3 heights were defined to measure the mean width. At 15 days, ten F1 plants per treatment from different pots were taken and the expression of defense and phytohormone crosstalk marker genes was analyzed by qPCR. At 21 days, the Tref substrate of five pots containing one remaining plant each was infected with 1,000 Mj eggs. At 51 days (30 dai with Mj), the five F1 plants per treatment were used to determine the NG, NEM and the NEM/NG ratio, and to calculate the green mass geometric parameters.

### Real-time PCR analysis

Gene expression was analyzed by qPCR. cDNA was synthesized from 1 μg of total RNA, which was extracted from pooled root samples of three different F0 plants or ten pooled F1 plants using TRIZOL^®^ (Invitrogen Life Technologies, Carlsbad, CA, USA), purified with RNeasy MinElute Cleanup kit (Qiagen, Hilden, Germany), and then used for reverse transcription with an oligo(dT) primer with the Transcriptor First Strand cDNA Synthesis kit (Takara Inc., Tokyo, Japan), following the manufacturer’s protocol. PCR reactions were performed using an ABI PRISM 7000 Sequence Detection System (Applied Biosystems, Applied Foster City, USA) with Brilliant SYBR Green QPCR Master Mix (Roche, Penzberg, Germany) in a total volume of 10 μl. F0 root cDNA obtained from two independent experiments (roots of three F0 plants per each treatment and time point), and F1 plant cDNA from two independent experiments (10 F1 plants per treatment) were used and three technical replicas were analyzed. All reactions were performed under the following conditions: an initial denaturation step (20 sec at 95 °C) followed by 40 cycles of denaturation (15 sec at 95 °C), annealing (1 min 60 °C), and extension (1 min 72 °C); and a final extension (3 min 72 °C). CT values were calculated using the Applied Biosystems software, and transcript abundance was calculated in Microsoft Excel from Ct (cycle threshold) values and normalized to the *actin* gene signal. The relative expression levels were calculated using the 2^−ΔΔCT^ method^56^. Marker genes representative of SA (*PR1, LECHS2* and *NPR1*), JA (*LOX1* and *MYC2*), and JA/ET (*ERF1*) defense pathways, as well as *TPX1, LERBOH1* and *ARF1* genes encoding a peroxidase specifically involved in lignification, a NADPH oxidase involved in oxidative burst and the TF auxin response factor 1, respectively, were analyzed by qPCR. The specific primers used are shown in Table S1^30^,^57^,^58^,^59^,^60^,^61^.

### Statistical analysis

The data were transformed, if necessary, to meet the statistical assumptions of normality^62^ and homogeneity of variances^63^. Then, the data set was submitted to analysis of variance and means compared by Tukey test (*P* < *0.05*) using Statistica 7 software (Statsoft Inc.).

## Additional Information

**How to cite this article**: Medeiros, H. A. *et al*. Tomato progeny inherit resistance to the nematode *Meloidogyne javanica* linked to plant growth induced by the biocontrol fungus *Trichoderma atroviride. Sci. Rep.* **7**, 40216; doi: 10.1038/srep40216 (2017).

**Publisher's note:** Springer Nature remains neutral with regard to jurisdictional claims in published maps and institutional affiliations.

## Supplementary Material

Supplementary Figure S1 and Table S1
