# Supplementary material for: Tomato progeny inherit resistance to the nematode Meloidogyne javanica linked to plant growth induced by the biocontrol fungus Trichoderma atroviride
Source: Sci Rep. 2017 Jan 10;7:40216. doi: 10.1038/srep40216 (PMC5223212; doi:10.1038/srep40216)
Supplement: Supplementary Figure S1 and Table S1 [file srep40216-s1.doc]

Tomato progeny inherit resistance to the nematode *Meloidogyne javanica* linked to plant growth induced by the biocontrol fungus *Trichoderma atroviride*

Hugo Agripino de Medeiros, Jerônimo Vieira de Araújo Filho, Leandro Grassi de Freitas, Pablo Castillo, María Belén Rubio, Rosa Hermosa, Enrique Monte^*^

**Supplementary information**


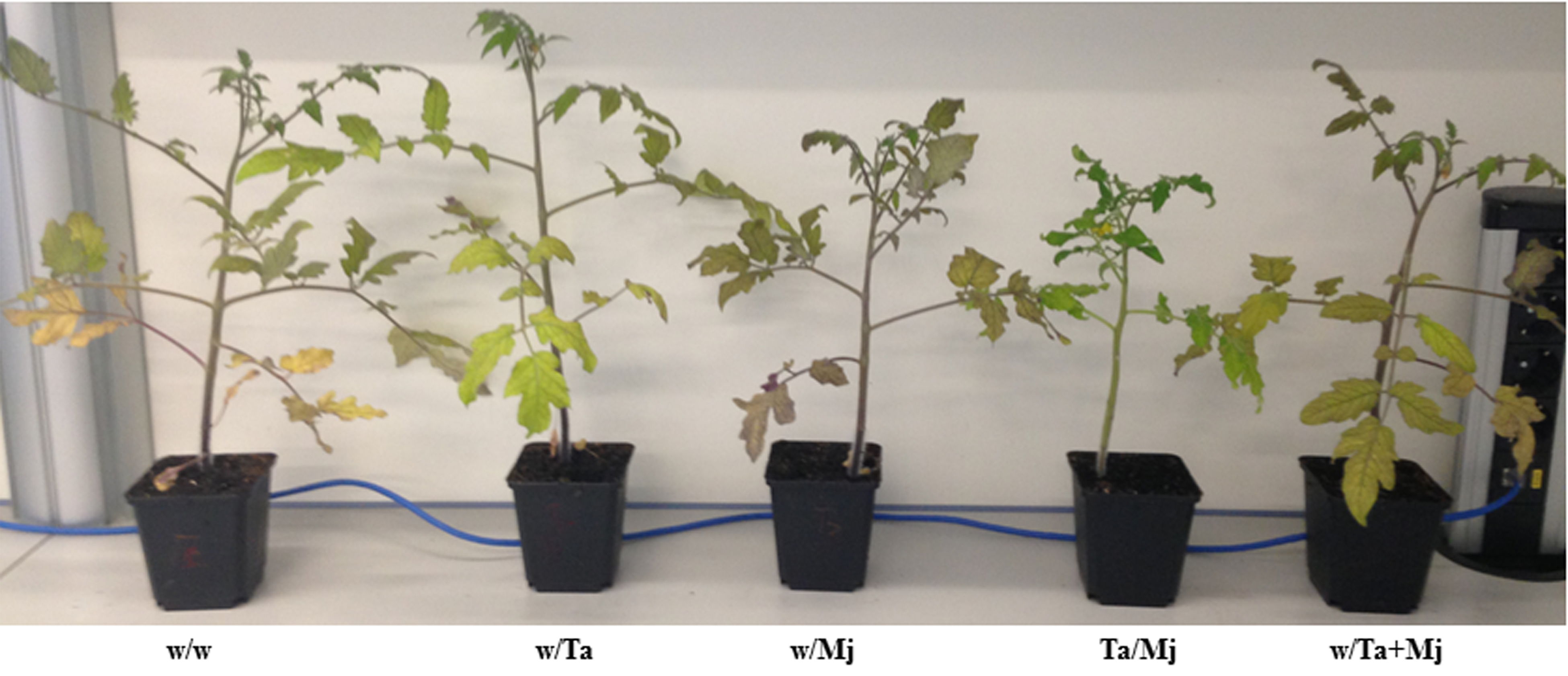


**Fig. S1. F1 tomato plants infected with *Meloidogyne javanica* (Mj).** Fifty-one-day-old F1 plants at 30 days after infection with Mj, when plants were 21-days old. These plants were derived from seeds collected from the following five split-root tests: i) (w/w) the two root system halves treated with sterile water (control); ii) (w/Ta) one root system half inoculated with *Trichoderma atroviride* T11 (Ta); iii) (w/Mj) one root system half inoculated with Mj; iv) (Ta/Mj) one root system half inoculated with Ta and the other half inoculated with Mj; and v) (w/Ta+Mj) one root system half inoculated with Ta plus Mj.

**Table S1. Primers used for quantitative Real-time PCR (qPCR) analysis.** Oligonucleotides used for qPCR analysis of tomato genes involved in defense responses.

| **Name** | **Sequences (5’ → 3’)** | **Gene** | **Reference** |
| --- | --- | --- | --- |
| PR1-fw1 | CCTCAAGATTATCTTAACGCTC | Pathogenesis-related (*PR1*) | 30 |
| PR1-rev1 | TACCATTGCTTCTCATCAACC |  |  |
| LOX f | GCCTCTCTTCTTGATGGA | Lipoxygenase (*LOX1*) | 30 |
| LOX r | GTAGTGAGCCACTTCTCCAA |  |  |
| TPX1-fw | GCTTTGTCAGGGGTTGTGAT | Peroxidase (*TPX1*) | 57 |
| TPX1-rev | TGCATCTCTAGCAACCAACG |  |  |
| LeRBOH1-fw | GTCAGGCTTCTACAGAAAAC | NADPH oxidase (*LeRBOH1*) | 58 |
| LeRBOH1-rev | GTTGATTACAGTAGCCGGTTC |  |  |
| LeCHS-2-fw | TTCGGTTAAGCGGCTCATGA | Chalcone synthase (*LeCHS-2*) | 59 |
| LeCHS-2-rev | CTCGAGCACCCTTGTTGTTCTC |  |  |
| MYC2-fw | CTGAAAAGAAGCCGAGGAAGC | Transcription factor (*MYC2*) | This work |
| MYC2-rev | GCATCTCCAAGAAGTGATGCC |  |  |
| ERF1-fw | AAGTGGCTCGCCTAAGAGGA | Ethylene response factor *(ERF1)* | 60 |
| ERF1-rev | TAACATTTGGTCCCCGGCTC |  |  |
| NPR1-fw | GGCGGACAACCTGCGTCAAC | Nonexpressor of pathogenesis-related gene *(NPR1)* | 61 |
| NPR1-rev | GCTCTCGTGGTCTGGCAAGC |  |  |
| SlARF1-fw | GCAGCAACACCTACAAC | Auxin response factor *(ARF1)* | This work |
| SlARF1-rev | ACAGGAGACTTCCACATTC |  |  |
| Actin-fw | CACCACTGCTGAACGGGAA | Actin (*ACT*) | 30 |
| Actin-rev | GGAGCTGCTCCTGGCACTTT |  |  |

Oligonucleotides used for qPCR analysis of tomato genes involved in defense responses.
